# Supplementary material for: Cervicovaginal Microbiota Profiles in Precancerous Lesions and Cervical Cancer among Ethiopian Women
Source: Microorganisms. 2023 Mar 24;11(4):833. doi: 10.3390/microorganisms11040833 (PMC10144031; doi:10.3390/microorganisms11040833)
Supplement: Supplementary file 1 [file microorganisms-11-00833-s001.zip › microorganisms-2225406-supplementary tables.pdf]

Supplementary Table S1

Histological/Cytological status of study participants based on age category.

	Isohelix TM DNA Buccal swab	Evalyn Brush
Cancer	60	0
Dysplasia	6	19
none	1	34

Supplementary Table s2

Histological/Cytological status of study participants based on type of sample collection device

	<50 years	>50 years
Cancer	28	32
Dysplasia	22	2
none	34	1
