# Supplementary figures and images for: Cervicovaginal Microbiota Profiles in Precancerous Lesions and Cervical Cancer among Ethiopian Women
Source: Microorganisms. 2023 Mar 24;11(4):833. doi: 10.3390/microorganisms11040833 (PMC10144031; doi:10.3390/microorganisms11040833)

**A**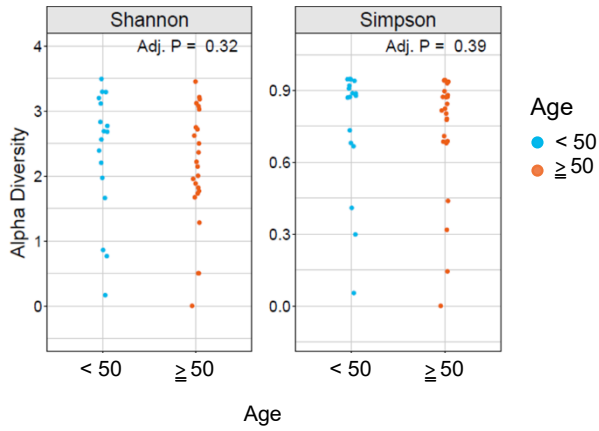**B**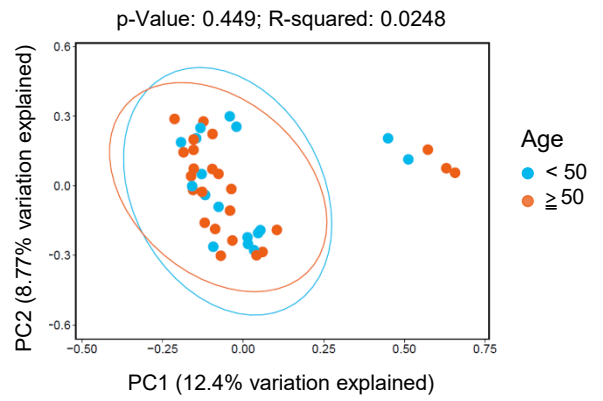**C**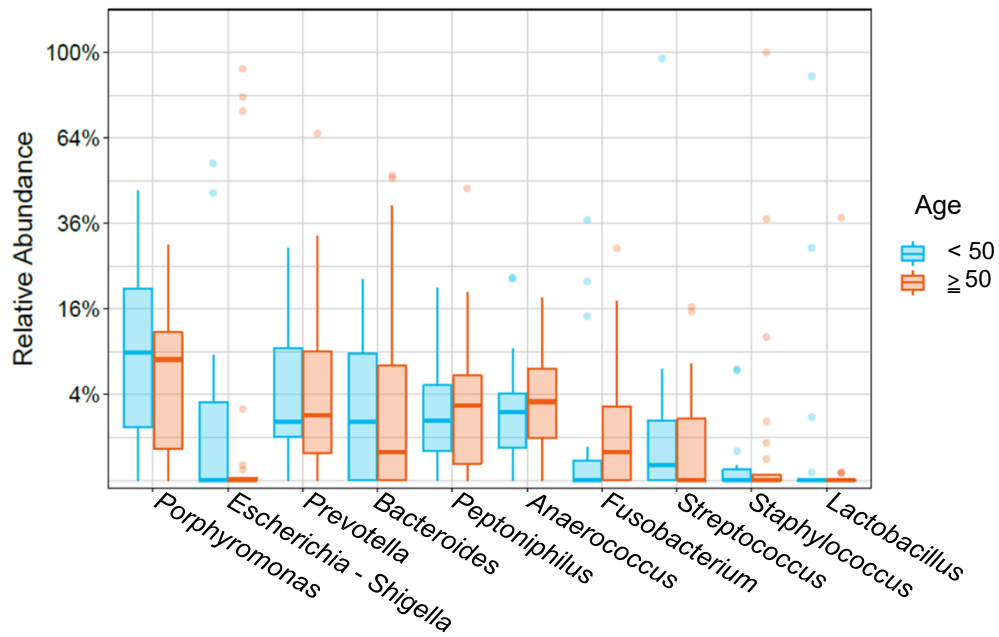

Supplement: Supplementary file 1 [file microorganisms-11-00833-s001.zip › Supplementary Figure S1.pdf]

A

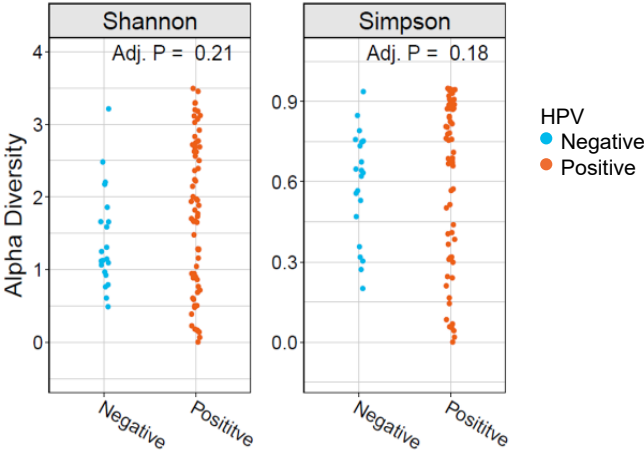

B

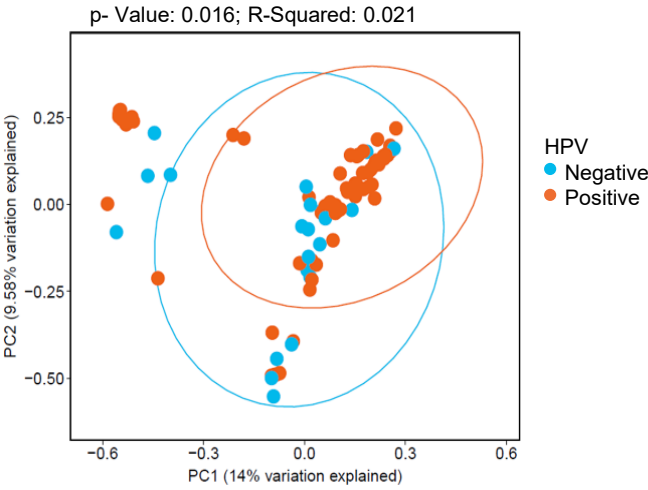

Supplement: Supplementary file 1 [file microorganisms-11-00833-s001.zip › Supplementary Figure S2.pdf]

A

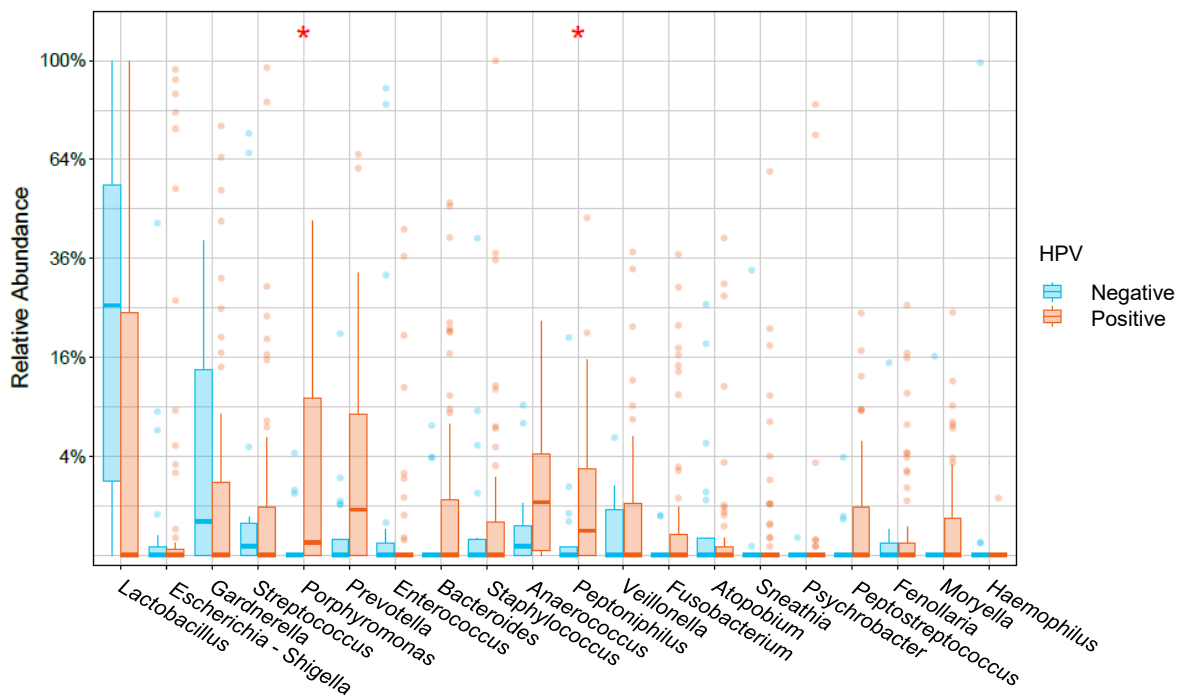

B

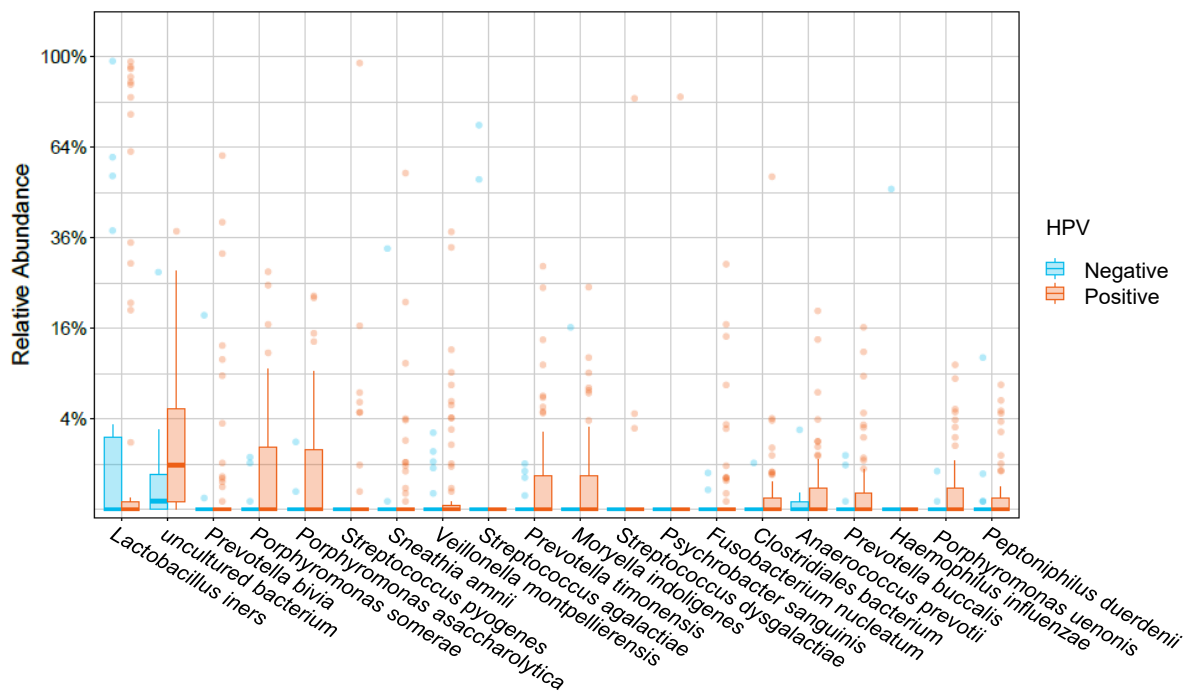

Supplement: Supplementary file 1 [file microorganisms-11-00833-s001.zip › Supplementary Figure S3.pdf]

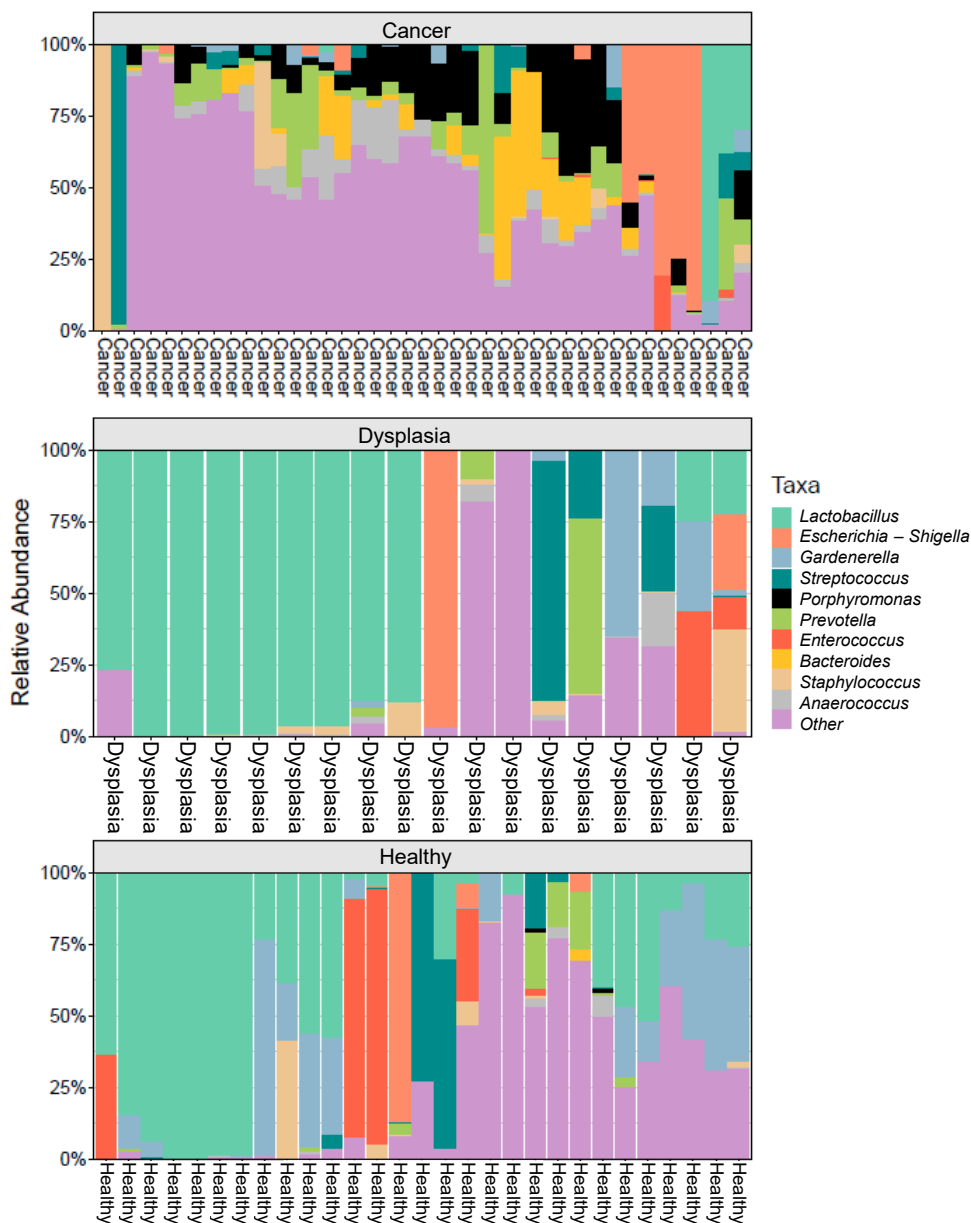

Supplement: Supplementary file 1 [file microorganisms-11-00833-s001.zip › Supplementary Figure S4.pdf]
